# Impact of Work–Family Conflict, Job Stress and Job Satisfaction on Seafarer Performance

**DOI:** 10.3390/ijerph17072191

**Published:** 2020-03-25

**Authors:** Ji An, Yun Liu, Yujie Sun, Chen Liu

**Affiliations:** 1 Merchant Marine College, Shanghai Maritime University, Shanghai 201306, China; anji@shmtu.edu.cn; 2 Yangshan Port Maritime Safety Administration, Shanghai 200080, China; sunyujie@shmsa.gov.cn (Y.S.); liuchen@shmsa.gov.cn (C.L.)

**Keywords:** work–family conflict, stress, seafarer, performance, safety

## Abstract

A lack of research has been undertaken to explore work–family conflict and its impact on the shipping industry. The objective of the present study was to empirically examine the effects of work–family conflict, job stress, and job satisfaction on seafarer performance. Data were collected from merchant ship seafarers in the Yangshan Port, Shanghai, China (*n* = 337). A data analysis was performed using hierarchical regression analysis. The research results revealed that work–family conflict and job stress negatively affects seafarer self-reported performance, while job satisfaction positively influences seafarer job performance. Findings also show that job satisfaction plays a moderating role in the relationships between work–family conflict, job stress and seafarer performance. Our findings demonstrate that work–family conflict, job stress and job satisfaction manifested are significant predictors for seafarer performance. Important applications and implications are provided for managers and researchers.

## 1. Introduction

Seafarer is considered to be a highly stressful [1] and high-risk occupation in terms of physical and mental exhaustion [2,3]. In fact, seafarers are easily subject to many psychosocial stressors and physical stressors, encompassing high leadership responsibilities (time pressure and decision-related pressure, under-qualified subordinate crew members), separation from family, loneliness, cross-cultural communication, fatigue and sleep deprivation, physical need, recreation scarcity, workplace noise, ship movement, vibration and heat [4,5,6,7,8,9]. 

Seafarers must sign long-term contracts with shipping companies, and specify how long they must stay on the ship (usually months or weeks), which means endured absence from home and society [4]. Among seafarers, the most frequently mentioned psychosocial stressors are long periods of separation from families and social isolation on shipboard [5,8,10]. Separation from family is the most prominent stressor for seafarers [4], and loneliness in the vessel was found to be associated with separation from family [5]. There is poor accommodation and a lack of recreational activities (the problem is more prominent when seafarers are on a long voyage), and these situations exacerbate the feeling of seafarer loneliness and alienation from the family [11]. For seafarers, work demands (i.e., separation from family) lead to the experience of work–family conflict (WFC), as individuals put forth more resources (such as time) to work, resulting in fewer resources being devoted to families [12]. Individuals will feel stressed when they lack the necessary resource to fulfill both work and family roles [13]. WFC refers to the pressure perceived by the individual when the family demands and the work demands are incompatible [14]. WFC has been identified as one of the major antecedents causing seafarer stress [15] and further been proved to damage individuals’ mental and physiological health [16]. To date, little attention has been paid to exploring the relationship between WFC and seafarer performance, and the effect of WFC on seafarer performance remains particularly understudied. In order to address this concern and plug this gap in the literature, WFC was introduced as one possible predictor of seafarer performance. 

Role theory is often used to explain the occurrence of WFC [17]. Based on the role theory, when the WFC intensifies, the consequent pressure leads to individual energy dissipation and distraction. Role theory shows that the pressure caused by role overload and role conflict will be detrimental to work outcomes [18]. Workers will not be able to effectively participate in work, because they possess fewer resources to engage in the work domain [19]. In this way, individual job performance may decrease as a result of resource depletion. Rather, the more efforts a person makes for satisfying work demands, the fewer efforts an individual puts forth to meet family requirements [20]. Within this framework, when the WFC increases, seafarers will not be able to effectively engage on the family field, which may be attributed to their work or organization. As a result, seafarers’ attitudes towards the organization or work will tend to be negative. As Carlson et al. [18] commented that, when an individual experiences anxiety originating from job role stress, then the one is more likely to be discontent with the work role. Stated alternatively, seafarers’ job satisfaction will decrease. In this light, staying away from family is considered by seafarers to be one of the major factors that reduce their motivation to work [21]. Seafarers may thus be absent from work more often, be less effective, make wrongdoing frequently, and have a stronger willingness to leave the ship.

Crew members are often exposed to high levels of physical and mental stress, which often persist for months [4,5,22]. Relationships with families and lifestyle change as a result of social isolation were deemed to be the most influential antecedent of perceived stress [23]. Job stress, in the current study, could be emerged in the surroundings (i.e., the workplace) or in work itself, and may emanate from role ambiguity, overworked, role conflicts, and time pressure. Seafarer job stress has been found to be linked to WFC [15] and psychoticism [23], and has been served as one of the major causes of maritime accidents [24] and seafarer burnout [25]. Stress leads to reduced job satisfaction [26] and hinders seafarers in performing their tasks effectively [27]. Many of the stressors faced by seafarers are long-term, and extended exposure to the marine surrounding causes higher stress [22,28]. Further, Slišković and Penezić [10] noted that the level of gastrointestinal symptoms of seafarers was higher under longer onboard duration and contract compliance. Since work stress increases health risk and reduces safety performance [24,29,30], long-term work stress can affect safe behavior and increase workplace injury risk [31,32]. It can be argued that a seafarer has to withstand great stress, resulting from long-time separation from family. Such stress may engender reduced job satisfaction, and subsequently performance.

Job satisfaction is considered a crucial concern in the shipping industry [33,34] and closely linked to safety in the maritime field [34]. When seafarers perceived high job satisfaction on a continual basis, seafarers’ motivation to conform to safety operation regulation increases [35], and seafarer performance is enhanced [36]. This impact has been associated with the safety among the maritime field [34], such as safety climate perception [37] and safety perceptions [38]. Specific research by some scholars further supports this relationship in other areas, such as miners [39], and company employee [40]. Besides, a great deal of studies has shown that reduced employee job satisfaction arising from work stress can result in increased absenteeism and intention to leave, as well as reduced productivity and job performance [41]. Using linked survey and register data, Böckerman and Ilmakunnas [42] pinpointed that dissatisfied employees tend toward absenteeism and low productivity. Furthermore, job satisfaction may be affected by WFC, as individuals may be dissatisfied with their organization or supervisor if work is considered to be a reason for spending too little time with family [43]. In this way, seafarers would tend to be dissatisfied, due to long-term separation from family. As a result, seafarer efficacy may be thus diminished and their job performance would be reduced accordingly.

Job performance is a critical variable in organizational behavior, higher in which indicates fewer mistakes been made. In the maritime field, it means the ship can maintain a normal working condition as a result of seafarers’ low human error. Job performance refers to the degree of an individual’s successful completion of task-related behaviors, and is an overall indicator of the individual’s performance in the course of working [44]. For seafaring occupation, it signifies quality and productivity (i.e., fewer human errors). Job performance is a critical work outcome that can be linked to individual factors of maritime personnel, including loyalty and work attitude [45], and job satisfaction [36]. Further, in the maritime field, seafarer performance has been found to be associated with safety perception [46], safety climate [47], and safety performance of ship operations [48]. The aforementioned factors play an essential role in seafarer psychological well-being and ship safety. The improvement of seafarers’ performance is beneficial for enhancing the turnaround time of ships, the safety performance of ship operations, and satisfying the efficiency requirements of ship-owners [48]. For shipping companies, this could save considerable costs and improve services.

Little was known about the effects of WFC in the maritime realm at the time we carried out this study; the present study fills one of these literature gaps and explicitly seeks to examine predictors of job performance in the maritime field. A review of previous research underscores the role of WFC in decreasing and lowering job performance [49]. Earlier studies have identified that WFC was associated with job performance [50]. According to Karatepe [51], WFC has an indirect effect on job performance. Nevertheless, an exploration of the impact of WFC on job performance in the stressful workplace environment of ship operation is lacking. To our best knowledge, the present study is one of the initiations to investigate the relationship between WFC, job stress, job satisfaction and seafarer performance in the seafaring context.

The purpose of this study is to fill the gaps in the literature by investigating several potential predictors of job performance, that are of particular relevance to seafaring: WFC, job stress, and job satisfaction. There were three aims in the present study. The first aim was to examine the effects of WFC, job satisfaction and job stress on seafarer self-reported performance. The second was to investigate whether job satisfaction and job stress play a moderating role in the relationship between WFC and seafarer performance. The third was to test the moderating effects of job satisfaction on the relationship between job stress and seafarer job performance.

## 2. Materials and Methods 

### 2.1. Sample and Data Collection

Before data gathering, all aspects of research had been approved by Merchant Marine College, Shanghai Maritime University, Shanghai, China. In addition, following a review procedure, further approval was received from the Shanghai Yangshan Port Maritime Safety Administration. Consent was obtained from all respondents. Cross-sectional data were gathered onboard through the questionnaire. Messages regarding research information were sent from the Port State Control (PSC) to the captains, requesting them to notify seafarer onboard finishing survey forms within the specified time. Respondents voluntarily filled out the anonymous paper-type questionnaires during ship berthing. Data for the number of seafarers on each ship who were informed of the research and asked to fill in the form were not available. Therefore, it is impossible to specify the response rate. The PSC did not exclude or select any ship or seafarer when issuing the questionnaires.

Participants were seafarers working on merchant ships. Merchant seafarers are divided into officers, engineers, ratings or crew, and catering. According to respective rank, these crews are further classified as follows, from captain to third officer, chief engineer to fourth engineer, caterings, and boatswain to general crew [52]. Among them, officers manage the navigation and the cargo operations, as well as mooring tools on the deck, while engineers are responsible for the maintenance and operation of machinery in the ship. Catering is mainly tasked with food preparation and ordinary housework on shipboard [53]. Data collection was conducted with the aid of PSC, the main idea of which is that when a ship sails to a foreign port, it can be inspected by the port’s state to ensure that the vessel complies with International Maritime Organization (IMO) standards [54]. PSC employees engage in the coordination and direction of the research, and reviewed the research design, procedure, analysis, and findings with the primary researchers. However, although the survey data collection was aided by the PSC, information, both personal and ship, will be most strictly treated for confidentiality, and the respondent identity cannot be known. Seafarers were voluntarily participating in this study, and withdrawal could be made at any moment without providing a reason. The survey data did not collect participants’ names, or correspondence information. Such procedures ensure the anonymity of the respondent. 

### 2.2. Analysis

We include and evaluate socio-demographic characteristics, three independents, and interaction terms in the current hierarchical regression analysis study. In model 1, race, rank, education, work experience, age, and marital status were entered. Within model 2, WFC was involved. Subsequently, job stress and job satisfaction were included in model 3. Interaction terms were added in the fourth model for assessing moderate effects. Job performance was the dependent variable for this study. 

The data screening procedures were completed prior to the regression analysis. Psychometric analyses and exploratory factor analysis (EFA) were included in preliminary analyses, which were also conducted. The proposed indicators of seafarer performance were examined by virtue of hierarchical multiple regressions. In the meantime, variables were included into the regression models in the designated sequence. Model 1 included socio-demographic characteristics which were specified as control variables. Then, we included WFC. Job satisfaction and job stress were entered in Model 3. In Model 4, we include interaction terms to assess the moderating effect. We checked the standardized coefficients, significance, and t-values, in order to evaluate the effects of predictors on job performance. In particular, the R^2^ of each model was calculated to examine whether the added model had a significant change compared to the previous model. The above-mentioned analysis was conducted using the SPSS (version 24, IBM Corporation, New York, USA).

### 2.3. Measures

#### 2.3.1. WFC

WFC was evaluated by a six-item scale developed by Matthews, Kath, and Barnes-Farrell [55]. This scale had been demonstrated to have good reliability and construct validity in the English context [56]. Items include, “I have to miss family activities due to the amount of time I must spend on work responsibilities”. ”I am often so emotionally drained when I get home from work that it prevents me from contributing to my family.” “The behaviors I perform that make me effective at work do not help me to be a better parent and spouse.” “I have to miss work activities due to the amount of time I must spend on family responsibilities.” “Because I am often stressed from family responsibilities, I have a hard time concentrating on my work.” and “Behavior that is effective and necessary for me at home would be counterproductive at work.” Items were evaluated on a 5-point Likert-type scale, which ranges from “1 = totally disagree” to “5 = totally agree”. Cronbach’s α for the scale was 0.90.

#### 2.3.2. Job Stress

Job stress was measured by a five-item scale developed by Fairbrother, K. and Warn, J. [57]. The decision to adopt this scale was grounded on its validity, acceptable internal consistency, and conciseness [36,57]. Items were evaluated using a 5-point Likert-type scale, which ranges from “1 = totally disagree” to “5 = totally agree”. The items encompass “There is insufficient work-life balance for me.” “There is insufficient co-workers’ support at work.” “There is insufficient shore-staff support at work,” “My working hours and work schedules are not well-planned.” and “The working and living conditions in ships are not acceptable.” Cronbach’s α for the present scale was 0.85.

#### 2.3.3. Job Satisfaction

Job satisfaction was evaluated on a two-item scale derived from Wanous et al. [58]. Yuen et al. [36] has further validated this scale for seafarers in the English context. Each item was assessed using a 5-point Likert-type scale, which ranges from “1 = totally disagree” to “5 = totally agree”. Scale items include “I am satisfied with my job.” and “I am satisfied with my company.” Cronbach’s α was 0.84.

#### 2.3.4. Job Performance

Job performance was measured using the three-item scale from Sánchez-Beaskoetxea and Coca García [59]. This scale was later adapted by Yuen et al. [36] for seafarers in the English context. The Cronbach’s α for this scale was 0.92. Items comprising the scale include “I am seldom absent from work.”, “I make few mistakes at work.” and “I complete my tasks efficiently.” Each item was evaluated on a 5-point Likert scale, which ranges from “1 = totally disagree” to “5 = totally agree”.

## 3. Results

### 3.1. Respondent Characteristic

The crew members involved varied in age between 19 and 64 (M = 36.80, SD = 9.75). Of the total 337 valid questionnaires, most respondents, 284 (84.3%) were Asian, including Chinese, Filipino, etc. 36 (10.7%) were European, including British, etc. and the remaining 17 were from other regions (see Table 1). In terms of rank, many of the participants were ratings or crews (48.4%). The rest of the sample consisted of officers (26.7%), engineers (19.9%), and those who reported their rank as caterings (5%). With respect to education, the majority of seafarers achieved a college degree (43.6%). Around a quarter of respondents (24.9%) reported that they accomplished university or above education; less than one-third of (31.5%), namely 106 participants, accomplish high school or below level education. With regard to the work experience, 54 (16%) participants had less than five years’ working onboard experience and only 12 (3.6%) had been seafarers for less than one year. Around one-fifth (19.9%) had worked onboard for more than 20 years, and remain 64.1% had maritime experience in range from 6 to 20 years. With regards to age, more than 40% (138) participants reported that their age was between 30 and 39 years, 24.6% between 18 and 29 years, and 32.9% between 40 and 59 years. In particular, only 1.5% (5) participants indicated that their age was over 59 years. Lastly, with regards to marital status, the vast majority of the participants indicated their marital status as married, including live with a partner (78.6%). The remainders were unmarried, including divorced, widowed, or single (21.4%). The demographic characteristics of participants are delineated in Table 1.

### 3.2. Preliminary Data Analysis

Table 2 displays descriptive statistics, the correlation matrix, and the Cronbach α value. Although for larger sample sizes, correcting normality is not a major issue [60], we have evaluated the skewness and kurtosis for latent variables, according to the procedure proposed by Kline [61]. None of these are problematic (see Table 2). Since these values are acceptable and it is believed that normality corrections are usually only required for small samples [60], further sampling methods for normality were thus not completed. 

An assessment of the underlying factor correlation matrix demonstrated a significant negative correlation between seafarer performance and WFC (*r* = −0.366, *p* < 0.05) and job stress (*r* = −0.557, *p* < 0.05). There was a significantly positive relationship between job satisfaction and seafarer performance (*r* = 0.217, *p* < 0.001). Reliability was examined through assessing Cronbach’s alpha of each scale used in the study. Each alpha showed high internal consistency. 

In order to verify the predictive indicators and guarantee the construct and factorial validity, EFA was undertaken with the principal axis factoring. The application of EFA to generate validity information was supported by Kane’s validity method [62]. Prior to the EFA, Kaiser–Meyer–Olkin (KMO) sampling adequacy measurements and Bartlett sphericity tests confirmed that the data were suitable for factor analysis. The KMO value is 0.88, which is articulated appropriate for factor analysis [63]. The result of the Bartlett’s test (χ^2^ = 1990.277, df = 200, *p* = 0.000) also conforms to the standard for conducting a factor analysis [64].

In comparison to principal component analysis, the principal axis factoring was adopted, since the purpose of the analysis is to examine potential variables and latent constructs that constitute factors. It is assumed that three independent factors will manifest and that each scale item will be properly loaded on its own factor. Meanwhile, Kaiser Normalization rotation method, the Promax with Kappa = 4, was employed. We applied the oblique method, given that this method of rotation can more realistically and accurately represent the relationship between constructs [65].

As alluded to earlier, based on the principle that the eigenvalue is greater than 1.0, a three-factor solution is obtained by factor analysis. This solution supported our predictor measures, as three separate factors manifested, and each item of each scale was loaded on its own factor. All of factors explained 69.76% of the variance in total. Factor 1, the eigenvalue of which is 5.62, accounted for 45.10% of the variance; Factor 2 explained 14.57% of the variance, with an eigenvalue of 1.85. Factor 3 explained 10.10% of the variance, with an eigenvalue of 1.08. The EFA results are shown in Table 3. The loading values of these factors are 0.40 or higher. There is no problem with the double loading of the predictors. Three factors that emerge as follows: Factor 1 is WFC, Factor 2 is job stress, and Factor 3 is job satisfaction.

### 3.3. Hierarchical Regression Analysis

Avoiding multicollinearity in regression analysis is essential [66]. To test multicollinearity, the Durbin–Watson value of each regression equation was calculated. As shown in Table 4, Durbin–Watson values ranged between 1.701 and 2.014, which suggested that the residuals were uncorrelated, and the autocorrelation problem was not posing an influence to our analysis [67]. None of the control variables were significantly entered into regression models. In model 2, WFC was included. There was a significant change towards model 1, F(1, 329) = 24.815, *p* = 0.029, leading to a pronounced improvement in R^2^ from 0.057 to 0.671. In this model, the coefficients of WFC were negative and significantly affect seafarer performance (β = −0.123, *p* < 0.05), which shows that WFC is negatively associated with seafarer performance. Model 2 reveals that WFC has a significant effect on the seafarers’ performance. With the addition of job satisfaction and job stress, the coefficients of WFC were negative and without significant impact on seafarer performance (β = −0.104, *p* > 0.05). Job stress had a significantly negative impact on seafarer performance (β = −0.063, *p* < 0.01). Job satisfaction had a significant influence on seafarer performance, the coefficients of which were positive (β = 0.163, *p* < 0.01). The significant effect still existed after entering job satisfaction and job stress to Model 3. This step, F(2, 327) = 23.138, *p* = 0.045, caused significant improvement in R^2^ to 0.688, which suggests the significant impact of job satisfaction and job stress on seafarer performance. 

In Model 2, WFC was significantly inversely related to job performance, β = −0.123, *t* = −2.194, *p* < 0.05. In Model 3, job stress, β = −0.063, *t* = −2.466, *p* < 0.01 was highly significant and negatively related to job performance. Job satisfaction, β = 0.163, *t* = 2.501, *p* < 0.01 was highly significant and positively associated with seafarer performance. WFC was inversely associated with job performance but was not significant (β = −0.104, *t* = −0.950, *p* > 0.05). Finally, we examined the interaction items of WFC and job stress, WFC and job satisfaction, job stress and job satisfaction. This step brings about a significant change, F(3, 324) = 36.382, *p* = 0.000, which signifies the presence of a significant interaction between WFC, job stress, job satisfaction and seafarer performance. Model 4 shows that the interaction between WFC and job stress (β = 0.111, *p* > 0.05) was positive but not significant. The interaction between WFC and job satisfaction (β = 0.185, *p* < 0.05) was positive and significant. The interaction term between job stress and job satisfaction (β = −0.280, *p* < 0.01) was significantly negative. This suggested that job satisfaction significantly moderates the impact of WFC and job stress on self-reported performance. However, the interaction between WFC and job stress (β = 0.111, *p* > 0.05) was positive, yet not significant. This finding would indicate that job stress was not moderating the effect of WFC on performance. The hierarchical regression analysis results are displayed in Table 4.

## 4. Discussion

Although previous studies have identified a range of predictors for seafarer performance, such as payment, job stress and job satisfaction [36,45], an exploration of the impact of WFC on seafarer performance is lacking. This study developed a theoretical model to interpret the performance of seafarers on board and verified this model empirically. It emphasized the substantial value of WFC, job satisfaction and job stress in elucidating seafarer performance in the ship. It elaborated the way that WFC affects seafarer performance, by combining job satisfaction and job stress. The finding also illustrated that job satisfaction moderates the impact of WFC, job stress on seafarer performance. As far as we know, this study first of all provides empirical evidence to demonstrate the importance of WFC in predicting performance in the seafarer’s realm. Further, this research plugs the gap in the literature, as there is currently a deficiency of research on WFC in the maritime industry.

In the present study, a hierarchical multiple regression analysis was used to investigate the influence of WFC, job stress, and job satisfaction on seafarer performance. The major findings derived from the present study are as follows. First of all, a significantly negative relationship between WFC and seafarer job performance was identified, when only socio-demographic characteristics and WFC were included in the regression model. This result is justified on the basis of ego depletion theory [68]. According to ego depletion theory, individuals would suffer the loss of primary resources in the face of WFC. The secondary resources might be lost if the internal resources are not replenished immediately, or the replenished resources are insufficient to recuperate reduced resources [69]. Therefore, if individuals cannot invest or obtain resources, they will be scant of resources to effectively adjust their emotions and behaviors [70]. In this case, a reduction in job performance is liable to occur [71]. 

Second, job stress was found to be negatively associated with seafarer performance, whereas job satisfaction was positively associated with seafarer performance in our sample. This finding lends support to the conclusion in the prior research [36]. The study by Riyadi [72] revealed that job stress is one of the essential predictors which might reduce the job performance. Work stress must be managed, as it can give rise to various counterproductive work behaviors [73] and cause a lower work contribution [74]. Meanwhile, employees who are satisfactory with their work tend to exert a positive impact on the organization [75] and devote maximum contributions to the organization [73]. Instead, Nurak and Riana [76] pinpointed that those who are in low job satisfaction are inclined to violate the organizational policiesand may subsequently result in counterproductive behaviors in work. 

Although WFC explains reductions in job performance [77,78], the relationship between WFC and job performance is inconsistent [77,79,80], which suggests the possibility of uncovered moderators [79,80,81]. Therefore, Model 4 was used to examine the moderating effect, which was significant, other than the interaction of WFC and job stress. The results of moderated regression analysis showed that job satisfaction positively moderated the impact of WFC on seafarer performance, whereas job satisfaction plays a negatively moderating role in the relationship between job stress and seafarer performance. 

### 4.1. Practical Implications

The findings of the present research have several paramount implications for ship resource managers to enhance seafarer performance and ship safety. First of all, since WFC is one of the crucial factors predicting seafarers’ job performance on board, it must be considered by ship resource managers. We notice that when WFC increases, seafarers’ performance can reduce. Moreover, seafarer performance is an essential contributor to ship safety [15]. Therefore, to enhance seafarers’ job performance and reduce job stress, WFC among seafarers should be a crucial concern. 

Ship resource managers should endeavor to implement flexible working arrangements [82], promote organizational support [83], and enhance leadership support [84], as these efforts may be demonstrated to be beneficial with reducing WFC and consequent stress, which we have identified as predictors of seafarer performance. Furthermore, interventions intended to bolster job resources, encompassing instrumental work support [28], and organizational justice [85], may be proven to be worthwhile, as these resources have been positively related to job satisfaction and negatively associated with job stress among seafarers. Ultimately, it is imperative to assess the effectiveness of these suggested interventions. We therefore recommended that future studies could explore the development, implementation and evaluation of these interventions.

More importantly, the present study shows that the influence of WFC interacts with job satisfaction; the impact of job stress interacts with job satisfaction. A noteworthy finding is that job satisfaction significantly moderates the influence of WFC and job stress on seafarer performance. This implies that when seafarers perceived lower job stress or increased job satisfaction on board, their performance could increase. 

### 4.2. Limitations and Future Directions

As with most research, the results of the present research must take into account certain limitations. Little research has investigated WFC manifestation in the seafaring, which connotes that the relevant literature foundation is insufficient. For preliminary analysis, we applied EFA in view of Kane’s validity method [62], to obtain information about validity. One might say that it is advisable to take confirmatory factor analysis (CFA) into consideration. We contend that this is inappropriate to designate a CFA model based on EFA results, as it is not suggested when adopting the identical sample [61]. Sample size in this research is not large enough for random segmentation to complete exploratory and confirmatory factor analysis. In future research, it will be beneficial to examine our findings in a larger group of seafarers. The relatively small sample also limits the generalizability of our findings. Future studies are therefore suggested to base their analysis on a larger sample, to improve the external validity of the results.

Secondly, since a response rate was impossible to specify, due to the fact that data concerning the number of seafarers who participated were not available, a sampling bias may therefore have possibly occurred. For instance, respondents who completed the questionnaire may be well behaved personnel (i.e., have good performance). In this light, we suggest that future researcher should ascertain the overall sample population, in order to obtain a response rate of random sampling.

Thirdly, while emphasizing that the information collected in research surveys will be treated with the most rigorous confidentiality and no one can be distinguished from the questionnaire, self-assessment performance data and participants’ views on stress or job satisfaction on board may be affected by self-serving bias, as workers are afraid of being accused by managers or PSC personnel and are thus reluctant to report accurately. Future studies could intentionally assess seafarers’ performance by managers’ rating. We recommend that further studies aim to identify how WFC, job stress and job satisfaction affect seafarer work outcomes (i.e., mistakes or injuries). Finally, the potentially causal relationships of WFC, job stress and job performance could not be examined because of the cross-sectional design. Future studies can therefore choose a longitudinal or experimental method to determine the relationships between WFC, job stress, and seafarer performance.

## 5. Conclusions

Overall, the performance of seafarers is a complex structure that may be manifested by various occupational factors. It could be thought that the predictors of job performance are of as equal importance to comprehend as job performance itself. Taken together, this study reminds the maritime organizations, researchers, and managers that seafarer performance is related to and predicted by WFC, job stress and job satisfaction. Our findings take another step towards revealing the complexity of job performance and its antecedents, especially in a highly stressful occupation such as seafarer. 

## Figures and Tables

**Table 1:** Demographics of participants.

Demographics	Frequency (*n* = 337)	Percentage (%)
Race		
Asian (Chinese, Filipino, etc.)	284	84.3
European (British, etc.)	36	10.7
Others (American, etc.)	17	5.0
Rank		
Officer	90	26.7
Engineer	67	19.9
Rating, Crew	163	48.4
Catering	17	5.0
Education		
University or above	84	24.9
College	147	43.6
High school or below	106	31.5
Work experience (Years)		
0–1	12	3.6
2–5	42	12.4
6–10	75	22.3
11–20	141	41.8
>20	67	19.9
Age		
18–29	83	24.6
30–39	138	41.0
40–59	111	32.9
59+	5	1.5
Marital status		
Married	265	78.6
Unmarried	72	21.4

**Table 2:** Internal consistency, descriptive statistics, and correlations.

	Work–Family Conflict	Job Stress	Job Satisfaction	Job Performance
Number of items	6	5	2	3
α	0.90	0.85	0.84	0.92
M	2.62	2.45	3.75	4.01
SD	0.67	0.81	0.95	0.62
Skewness	0.317	0.302	0.874	0.354
Kurtosis	−0.294	−0.309	0.672	0.365
Work–family conflict	1			
Job stress	0.679 *	1		
Job satisfaction	−0.445 **	−0.486 **	1	
Job performance	−0.366 *	−0.557 *	0.217 **	1

Note: * *p* < 0.05, ** *p* < 0.001.

**Table 3:** Exploratory factor analysis results.

Measures	Factor 1	Factor 2	Factor 3
I have to miss family activities due to the amount of time I must spend on work responsibilities.	0.758	0.175	0.033
I have to miss work activities due to the amount of time I must spend on family responsibilities.	0.494	0.002	0.257
I am often so emotionally drained when I get home from work that it prevents me from contributing to my family.	0.705	0.077	0.187
Because I am often stressed from family responsibilities, I have a hard time concentrating on my work.	0.696	0.214	0.085
The behaviors I perform that make me effective at work do not help me to be a better parent and spouse.	0.743	0.011	0.366
Behavior that is effective and necessary for me at home would be counterproductive at work.	0.669	0.201	0.288
There is insufficient work-life balance for me.	0.056	0.689	0.126
There is insufficient co-workers’ support at work.	0.321	0.701	−0.261
There is insufficient shore-staff support at work.	0.083	0.741	−0.272
My working hours and work schedules in the ship are not well-planned.	0.178	0.636	−0.330
The working and living conditions in ships are not acceptable.	−0.007	0.758	−0.264
I am satisfied with my job.	−0.027	0.030	0.524
I am satisfied with my company.	0.114	0.095	0.701
Eigenvalue	5.62	1.85	1.08
Percentage of variance	45.10	14.57	10.10
Acuminated percentage	45.10	59.67	69.76

Note: Extraction Method: Principal Axis Factoring. Rotation Method: Promax with Kaiser Normalization.

**Table 4:** The results of hierarchical multiple regression analysis.

Model and Predictors	β	p	R^2^	Adjusted R^2^	F	Significance of F	Durbin–Watson
Model One			0.057	0.040	3.328	0.003	1.857
Race	−0.187	0.051					
Rank	−0.063	0.296					
Education	0.084	0.157					
Work experience	0.029	0.702					
Age	−0.164	0.056					
Marital status	−0.110	0.089					
Model Two			0.671	0.651	24.815	0.029	1.753
Race	−0.194	0.132					
Rank	−0.071	0.238					
Education	0.112	0.066					
Work experience	0.018	0.243					
Age	−0.169	0.131					
Marital status	−0.116	0.073					
Work–family conflict	−0.123 **	0.029					
Model Three			0.688	0.663	23.138	0.045	1.701
Race	−0.196	0.068					
Rank	−0.073	0.223					
Education	0.144	0.019					
Work experience	0.024	0.751					
Age	−0.192	0.074					
Marital status	−0.122	0.058					
Work–family conflict	−0.104	0.064					
Job stress	−0.063 ***	0.003					
Job satisfaction	0.163 ***	0.009					
Model Four			0.639	0.607	36.382	0.000	2.014
Race	−0.196	0.131					
Rank	−0.073	0.214					
Education	0.116	0.056					
Work experience	0.019	0.907					
Age	−0.186	0.115					
Marital status	−0.122	0.051					
WFC	−0.968 ***	0.004					
JST	0.475	0.200					
JSA	0.168 **	0.014					
WFC × JST	0.111	0.055					
WFC × JSA	0.185 **	0.012					
JST × JSA	−0.280 ***	0.000					

Note: ** *p* < 0.05; *** *p* < 0.01; WFC: work–family conflict; JST: job stress; JSA: job satisfaction.
